# Robust Economic Control Decision Method of Uncertain System on Urban Domestic Water Supply

**DOI:** 10.3390/ijerph15040649

**Published:** 2018-03-31

**Authors:** Kebai Li, Tianyi Ma, Guo Wei

**Affiliations:** 1 School of Management Science and Engineering, Nanjing University of Information Science & Technology, Nanjing 210044, China; matianyi0820@163.com; 2 Department of Mathematics and Computer Science, University of North Carolina at Pembroke, Pembroke, NC 28372, USA; guo.wei@uncp.edu

**Keywords:** water management, uncertain system, robust control decision

## Abstract

As China quickly urbanizes, urban domestic water generally presents the circumstances of both rising tendency and seasonal cycle fluctuation. A robust economic control decision method for dynamic uncertain systems is proposed in this paper. It is developed based on the internal model principle and pole allocation method, and it is applied to an urban domestic water supply system with rising tendency and seasonal cycle fluctuation. To achieve this goal, first a multiplicative model is used to describe the urban domestic water demand. Then, a capital stock and a labor stock are selected as the state vector, and the investment and labor are designed as the control vector. Next, the compensator subsystem is devised in light of the internal model principle. Finally, by using the state feedback control strategy and pole allocation method, the multivariable robust economic control decision method is implemented. The implementation with this model can accomplish the urban domestic water supply control goal, with the robustness for the variation of parameters. The methodology presented in this study may be applied to the water management system in other parts of the world, provided all data used in this study are available. The robust control decision method in this paper is also applicable to deal with tracking control problems as well as stabilization control problems of other general dynamic uncertain systems.

## 1. Introduction

According to China’s regulations, “urban” is defined as locations of county authorities or higher level authorities, as well as locations that have permanent resident populations of between 2000 and 100,000, among which the non-agricultural population accounts for more than 50% of the residential areas.

China is undergoing a large-scale urbanization with a rapid growing urban population. Consequently, urban domestic water demand is increasing dramatically. In addition, urban domestic water often exhibits obvious characteristics of seasonal cycle fluctuation. Concerning this kind of characteristics of urban domestic water demand, we investigate the robust control decision method for water supply systems.

### 1.1. Background

Urban water generally includes residential domestic water, industrial water, commercial water and public water (Baumann et al. [1]). According to “The China water resources bulletin”, demographic data issued by the Ministry of Water Resources of China, and National Bureau of Statistics of China, China’s total annual domestic water consumption was 57.5 billion m^3^ in 2000, 67.5 billion m^3^ in 2005, 76.6 billion m^3^ in 2010 and 79.4 billion m^3^ in 2015, which accounts for, respectively, 10.5%, 12.0%, 12.7% and 13.0% of the annual total water consumption. China’s urban domestic water consumption was 36.7 billion m^3^ in 2000, 43.3 billion m^3^ in 2005, 47.2 billion m^3^ in 2010 and 61.1 billion m^3^ in 2015, which accounts for, respectively, 63.8%, 64.1%, 61.6% and 76.9% of the annual total domestic water consumption. This is the total water consumption for both residential use and for public use. Water sources include all sources, such as boreholes, aquifers, water reuse, etc.

According to “The China’s urban water supply bulletin (2006–2010)”, issued by the Ministry of Housing and Urban-rural Development of China, China’s urban water supply coverage rate, namely the proportion of the urban population that are provided with water supply, was 81.1% in 2005 and 90.3% in 2010.

According to “The China’s urban drainage and sewage treatment bulletin (2006–2010)”, issued by the Ministry of Housing and Urban-Rural Development of China, in 2010, the treatment capacity of urban sewage reached 125 million m^3^/day, which was 80% higher than that in 2005. The total amount of sewage treated in the year reached 35 billion m^3^. The production capacity of reclaimed water in urban sewage treatment reached 12.09 million m^3^/das, which was 10% of the scale of urban sewage treatment in China.

Along with the economic development and population growth, urban domestic water consumption is increasing, and its proportion in the total domestic water consumption is also rising. This reflects the present rapid urbanization of social development in China. Usually, the proportion of domestic water consumption in the total water consumption is positively related with the level of economic development.

According to the statistics for China’s eastern, central and western areas, from the east of China to the west, the domestic water ratio and industrial water ratio decrease gradually, but the proportion of agricultural water increases gradually, reflecting the situation that, in eastern China, there is more industry while in the western there more agriculture.

For Beijing, Chongqing and Tianjin, three Chinese municipalities directly under the central government, due to faster urbanization, the proportions of domestic water consumption in their total water consumption have exceeded 20%, a rate significantly higher than most other cities in China.

### 1.2. Literature Review

In 2011, Moreau et al. presented a control strategy for water heaters that minimizes the pick-up demand when the heating elements are reactivated at the end of a load shifting period and that ensures, in all cases, the client’s hot water supply; the study was based on a simulation model of a water heater that was experimentally validated and takes into account the diversity of the population’s hot water withdrawal profile [2]. In 2016, Pereira et al. presented a robust predictive controller for tracking periodic references to a section of Barcelona’s drinking water network, where the control objective was to satisfy the water demand while trying to follow a given reference of the level of the tanks in the network [3].

In 2013, Fernández-Seara et al. conducted an experiment to test the performance of a space/water heating system using an on demand external domestic hot water (DHW) production system (DHWPS) with different control strategies; results show that the control strategy of the DHWPS significantly influences the quality of the DHW supply and the thermal performance of the storage tank [4]. In 2014, Vansovits et al. designed a model predictive controller to identify industrial water boiler systems during a heating season, and its performance was compared with performance of classical proportional-integral (PI) control algorithm [5]. In 2015, an improved linear optimization model coupled with a computational iterative procedure of optimal discharges through pipes was developed by Sarbu et al. on the basis of linear programming for the design of new or partially extended water distribution networks, and the proposed model was compared to some other models [6]. In 2015, Sun et al. used a model predictive control as an integrated simulation and optimization modeling approach to provide the optimal configuration for large-scale water supply systems in real time, and conducted a case study based on the Catalunya regional water network approach [7].

Artificial neural network modeling method also has application in water management. In 2007, Al-Alawi et al. discussed a development of a predictive artificial neural network (ANN)-based prototype controller for the optimum operation of an integrated hybrid renewable energy-based water and power supply system (IRWPSS) [8]. in 2018, Nguyen et al. provided infrastructure planners with a detailed understanding of how granular data generated by an intelligent water management system (Autoflow^©^) can be utilized to obtain significant efficiencies throughout different stages of an urban water cycle, from supply, distribution, customer engagement, and even wastewater treatment [9]. In 2017, Sahoo et al. developed a new ensemble modeling framework based on spectral analysis, machine learning, and uncertainty analysis, as an alternative to complex and computationally expensive physical models. The new approach was applied and evaluated in the context of two aquifer systems supporting agricultural production in the United States: the High Plains aquifer (HPA) and the Mississippi River Valley alluvial aquifer (MRVA). [10]. In 2017, Qaderi et al. employed the statistical data concerning the groundwater quality during a 13-year period in Bareh Bijeh located in Ilam, Iran, to propose a new artificial neural network model for predicting the costs of water treatment through reverse osmosis [11]. In 2017, Sattar et al. developed a novel failure rate prediction model by the extreme learning machine (ELM) to provide key information needed for optimum ongoing maintenance/rehabilitation of a water network, meaning the estimated times for the next failures of individual pipes within the network [12].

Solar domestic hot water heating systems perform more efficiently if their storage tanks are perfectly thermally stratified. In 1992, Csordas et al. used a computer simulation to evaluate and compare two strategies by which plume entrainment is minimized by controlling the collector flow rate [13]. In 2001, Prud’homme et al. proposed and validated a predictive control strategy to improve the overall efficiency of solar kits for domestic hot water supply; this control strategy requires solving of an optimization problem and implementation of a state estimator [14]. The settings on the controller for a solar domestic hot water system can have major impact on the “ratings” obtained from a onw-day test or simulation. In 1994, Beckman et al. developed equations for optimal controller settings that will maximize the simulated performance [15]. In 2014, Mongibello et al. presented a transient numerical model for the simulation of a solar Domestic Hot Water (DHW) system. Their study was focused on the numerical simulation of a solar DHW system consisting of two flat plate collectors connected in series, a vertical cylindrical water tank for the heat storage, and a coil heat exchanger immersed into the water tank [16]. 

In 2001, Mourad et al. dealt with the application of adaptive control to a freshwater supply system; the main control objective was to regulate the consumption of water-flow by controlling the water pumps discharge and the adaptive control implemented was based on the linear quadratic control approach [17]. In 2003, Orlowski et al. discussed the objectives and various methods of implementing stepless control of pumps operating in water supply and sewage systems. Their analyses and conclusions presented are based on computer flow simulations in selected real and hypothetical systems [18]. Also in 2003, Eker et al. discussed the simulation and control of a water supply system including a series of pumping stations. The whole system was simulated and the results were presented and compared with the real-time measured data [19]. Heat pump systems have been widely used in buildings and industries due to their high performance. In 2013, Choi et al. proposed a leaving water temperature control scheme for a water-to-water heat pump for hot water supply [20].

The above research is largely concentrated on water heater or pump control engineering. Some researchers have studied the economic efficiency of water systems, as discussed below.

Cost-benefit analysis was used to investigate the economic viability of the proposed Melamchi water supply project (MWSP) for supplying water to the urban populations of the Kathmandu Valley in Nepal. In 2004, Whittington et al. utilized some Monte Carlo simulations to explore the sensitivity of the net present value and economic internal rate of return calculations to a wide range of assumptions and input parameters [21]. In 2010, to reconstruct past system behavior and diagnose the causes of a major water crisis in Chennai, India, Srinivasan et al. developed a hydrologic-engineering-economic model for addressing the complexity of urban water supply arising from consumers’ dependence on multiple interconnected sources of water. In this model, the Consumer Module is a microeconomic model and it simulated consumers’ decision-making processes by maximizing consumer surplus [22]. In 2010, Li et al. developed an economic optimal control model of urban water supply and demand discrete system’s investment [23]. In 2011, Li developed an economic optimal control model of town water supply investment based on dynamic share coefficient method [24]. In these models, investment is the sole control variables. In 2017, Wang et al. addressed a robust periodic economic model predictive control (EMPC) based on interval arithmetic with unknown-but-bounded additive disturbances for the management of water distribution networks (WDNs), and the periodically optimal steady states were obtained by employing a periodic EMPC planner with the nominal model [25].

### 1.3. The Problem

From the literature review in Section 1.2, nearly no attention has been paid to studying both investment and labor as control vector to the robust control decision of water supply systems. However, economic control decision is an equally important aspect for achieving the optimal operation of the urban water management system. As pointed out by Baumann et al. in 2005, considering from the perspective of economic value, the urban water industry is a capital-intensive industry without controversy [1]. Therefore, a robust economic control decision of urban domestic water systems with investment and labor comprises the main contents of this paper.

The motivation of this paper is that China’s rapid urbanization and urban population growth leads to the growth of the urban domestic water demand. At the same time, due to factors such as climate impact, the parameters of the urban domestic water system can fluctuate. To solve the urban domestic water demand problem in this situation, by determining the appropriate two kinds of factors of production in water supply (investment and labor input, and application of robust economic control decision method), the water supply can meet the domestic water demand, and the control scheme has robustness for system parameters fluctuation.

### 1.4. Structure of the Paper

In 2008, Pumphrey et al. conducted a survey on the urban and rural opinions or preferences toward municipal water controls for studying which policies would be most accepted among rural and urban communities in a semi-arid region of Texas, United States [26]. As urbanization is the trend of the social development in China, and domestic water is the premise of urban production and life, it is meaningful to safeguard the domestic water to meet the demand, and to optimize its supply with economic control method.

For this purpose, in this paper, we propose a dynamic model for the urban domestic water supply including rising tendency and seasonal cycle fluctuation, and solve it with the robust economic control decision method.

In Section 2, to reflect demographic factors and climate variables, we construct the expected dynamic model of urban domestic water supply by including rising tendency and seasonal cycle fluctuation. In Section 3, we devise the compensator subsystem and implement multivariable robust economic control scheme. In Section 4, we provide a numerical example for illuminating and validating the applications of the control scheme. In Section 5, compared with the existing models, from the perspective of economic control, our model studies the robust control of capital intensive urban domestic water system, and fills in a gap in the existing designs—lack of a utilization of multivariable robust economic control. Finally, Section 6 is devoted to summaries and conclusions.

Contributions to the water resource management by this paper include: establish a dynamic model of urban water supply and demand under the background of rapid urbanization. The multivariable robust economic control method for investment and labor input is used for urban water supply management, which fills in a gap in the existing designs in this field. When the parameters of the system are available, this method may also be applied to water management systems in other parts of the world. The robust control decision method in this paper is also applicable to deal with tracking control problems as well as stabilization control problems of other general dynamic uncertain systems.

## 2. Dynamic Model of Urban Domestic Water Supply

There are two kinds of models that can describe the demand function simultaneously including the rising tendency and the seasonal cycle fluctuation. The first is the additive model, and the second is the multiplicative model. The additive model is suitable for the situation where the seasonal cycle fluctuation is smooth and does not obviously change along with the rising tendency, while the multiplicative model is suitable for the situation where the seasonal cycle fluctuation is not smooth and obviously change along with the rising tendency (Jian et al. [27]).

Owing to demographic factors and climate variables, urban domestic water generally presents the circumstances of both rising tendency and seasonal cycle fluctuation. The rising tendency and the range of the seasonal cycle fluctuation of urban domestic water are usually positive correlative with the urban population. As China’s urbanization rate and urban population is rising (Wang et al. [28]), and there is a similar situation in other developing countries (Kallis et al. [29]), it is proper to adopt the multiplicative model for representing the urban domestic water demand function D(t), as follows:

(1) $$D(t)=d_{0}{\left(1+a\right)}^{t}\cos\beta t+d_{1}$$

where t is the continuous-time variable, a is the rate of urban domestic water demand rise whose constant value is known, ${\left(1+a\right)}^{t}$ represents the rising tendency of demand, β is the radian frequency whose constant value is known, cosβt represents the seasonal cycle fluctuation of demand, and $d_{0},d_{1}$ are coefficients whose constant values are unknown.

The rising tendency and seasonal cycle fluctuation relate in the multiplicative model. At present and in the quite long period of future, with the urbanization rate’s rapid increase in China, the urban population also continues growing, the urban domestic water demand shows the long-term rising tendency and expanded seasonal cycle fluctuation (Yin et al. [30]).

Equation (1) is a time series model. This kind of model is to attribute all the influencing factors to the time factor. It is believed that the comprehensive effect of all influencing factors will still play a role in the forecasting object in the future, and the influence of other factors is embodied in the time factor (Liu, [31]).

For example, the pricing structure is the influencing factor of the urban domestic water demand, and its impact on water demand can be reflected by Equation (1). When there is only a slight change in price, the impact on water demand may not be obvious, and the value of a remains unchanged. When the price rises more, it may inhibit the growth of water demand, and the value of a will become smaller. When the price rises significantly, the growth of water demand may be significantly suppressed, and the value of a becomes even smaller, even taking a negative value, which means that water demand is decreasing due to price adjustment.

To meet the urban domestic water demand, the urban domestic water supply industry needs to organize water production. On the basis of the supply theory of water resources economics (Spulber et al. [32]), yield is chiefly determined by the capital element and the labor element. Subsequently, the urban domestic water supply function S(t) can be represented as follows:

(2) S(t)=θK(t)+λL(t)

where K(t) is the capital stock, θ is the capital-output factor, L(t) is the labor stock, and λ is the labor-output factor. In consideration of the economic value of urban water industry, one thing that is rather noticeable is that, although the urban water industry has many absolute conditions, an exception is the aspect of capital-intensive. For example, in the United States, for the fiscal year 1993, the total water industry had about $41 billion in revenue, yet it gained $17.5 billion investment, which is 43% of the gross income after the audit. In the United States, no other major industrial department achieved such annual investment ratio (Baumann et al. [1]). Similarly, the urban water supply industry in other countries can also be treated as the representative of capital-intensive industries.

The influence of governance and ownership models of urban domestic water supply provision on Equation (2) can be reflected by the different values of θ and λ. The production of urban water supply is related to the efficiency of the use of capital and labor. When capital utilization is high, θ is high. When labor productivity is high, λ is high. In China, the urban water supply is the public service industry, and urban domestic water supply enterprises belong to state-owned, which is invested and operated by state-owned capital. In other countries, the value of θ and λ may be different if the governance and ownership of the urban domestic water supply company is different from that of China, such as the private sector.

The influence of the natural properties of water resources in Equation (2) can also be reflected by the different values of θ and λ. When water resources are easy to be exploited and used, for example, when the water source comes from surface water and less polluted, θ and λ may take larger values. When water resources are difficult to be exploited and used, such as when the water source comes from deep groundwater or recycled water, θ and λ may take smaller values.

On the basis of the theory of capital investment and human capital (Romer et al. [33]), the capital stock K(t) and the labor stock L(t) of urban domestic water supply can be represented as follows:

(3) K(t+1)=(1−δ)K(t)+μI(t)

(4) L(t+1)=(1−γ)L(t)+σP(t)

where I(t) is the amount of capital invested, μ is the rate of capital formation, δ is the rate of capital depreciation, P(t) is the amount of tyro in the industry, σ is the rate of labor formation, and γ is the rate of labor separation. Any tyro turns into the efficient labor just after professional training and producing practice.

Let

(5) Y(t)=S(t)−D(t)

where Y(t) is the urban domestic water supply imbalance with the demand.

Incorporating Equations (1–5) into the simultaneous equations, and by letting α=1+a, the model of urban domestic water system including the rising tendency and the seasonal cycle fluctuation can be represented as follows:

(6) $$\left\{ \begin{array}{l} D(t)=d_{0}\alpha^{t}\cos\beta t+d_{1}\\ S(t)=\theta K(t)+\lambda L(t)\\ Y(t)=S(t)-D(t)\\ K(t+1)=\left(1-\delta \right)K(t)+\mu I(t)\\ L(t+1)=\left(1-\gamma \right)L(t)+\sigma P(t) \end{array}\right.$$

In the control system, the stability index is often used as a measure whether the system can be long-term operated, and stability is the premise for the system to work regularly for a long time (Fang et al. [34]). From the point of view of systems stability, water resources security supply and efficient utilization, we set the system’s control goal to be asymptotic stable, and the urban domestic water supply imbalance with the demand Y(t) to converge to the desired value as follows:

(7) $$Y^{*}(t)=b$$

where b is a positive known number.

## 3. Robust Economic Control Decision Method

By substituting Equations (1) and (2) into Equation (5), Equation (6) can be simplified as follows:

(8) $$\left\{ \begin{array}{l} K(t+1)=\left(1-\delta \right)K(t)+\mu I(t)\\ L(t+1)=\left(1-\gamma \right)L(t)+\sigma P(t)\\ Y(t)=\left(\theta K(t)+\lambda L(t)\right)-\left(d_{0}\alpha^{t}\cos\beta t+d_{1}\right) \end{array}\right.$$

Thus, the system in Equation (8) is the tracking problem. In this problem, ${\left(K(t),L(t)\right)}^{T}$ is the two dimensional state vector, ${\left(I(t),P(t)\right)}^{T}$ is the two dimensional control vector, Y(t) is the one dimensional output variable, and D(t) is the one dimensional interference input variable. We expect, by means of assignment on the control vector ${\left(I(t),P(t)\right)}^{T}$ the output variable Y(t) to converge to the desired value $Y^{*}(t)$.

We will realize this control goal using the following steps.

Firstly, according to the internal model principle (Zhang [35]), we devise the compensator subsystem of the system in Equation (8). At the request of the internal model principle, the eigenvalues of the compensator subsystem should be consistent with that of the interference input variable D(t) and the desired output value $Y^{*}(t)$.

Secondly, we combine the state equations in the system in Equation (8) and the compensator subsystem. They take the form of a new matrix dynamic state equation.

Thirdly, we carry out the pole allocation on the above new matrix dynamic state equation through state feedback on control vector.

With the above procedures, our urban domestic water supply control goal is achievable and with robustness for the variation of parameters.

Now, we carry out the above procedures. 

First, we survey the eigenvalue of the interference input variable D(t) and the desired output value $Y^{*}(t)$.

Considering Equation (1), the *Z*-transform of D(t) is as follows:

(9) $$\begin{array}{ll} Z[D(t)] & =Z\left[d_{0}\alpha^{t}\cos\beta t+d_{1}\right]\\ & =Z\left[d_{0}\alpha^{t}\cos\beta t\right]+Z\left[d_{1}\right]\\ & =\frac{d_{0}\left(z^{2}-z\alpha \cos\beta \right)}{z^{2}-2z\alpha \cos\beta +\alpha^{2}}+\frac{d_{1}z}{z-1}\\ & =\frac{d_{0}z\left(z-1\right)\left(z-\alpha \cos\beta \right)+d_{1}z\left(z^{2}-2z\alpha \cos\beta +\alpha^{2}\right)}{\left(z-\alpha \left(\cos\beta +i\sin\beta \right)\right)\left(z-\alpha \left(\cos\beta -i\sin\beta \right)\right)\left(z-1\right)} \end{array}$$

Equation (9) shows that the eigenvalues of the *Z*-transform of D(t) are, respectively, α(cosβ+isinβ),α(cosβ−isinβ),1.

Considering Equation (7), the *Z*-transform of $Y^{*}(t)$ is as follows:

(10) $$Z\left[Y^{*}(t)\right]=Z\left[b\right]=\frac{bz}{z-1}$$

Equation (10) shows that the eigenvalue of the *Z*-transform of $Y^{*}(t)$ is 1.

Owing to the eigenvalues of D(t) and $Y^{*}(t)$ being solved, and considering the internal model principle, we can devise the compensator subsystem of the system in Equation (8) as follows:

(11) $$\xi_{1}\left(t+1\right)-\xi_{1}\left(t\right)=Y\left(t\right)-Y^{*}\left(t\right)$$

(12) $$\xi_{2}\left(t+2\right)-2\alpha \cos\beta \xi_{2}\left(t+1\right)+\alpha^{2}\xi_{2}\left(t\right)=\xi_{1}\left(t+1\right)$$

(13) $$\xi_{3}\left(t+2\right)-2\alpha \cos\beta \xi_{3}\left(t+1\right)+\alpha^{2}\xi_{3}\left(t\right)=\xi_{1}\left(t\right)$$

The eigenvalues of the homogeneous difference equations corresponding to Equations (11–13) are as follows: For Equation (11), it is 1, and for Equations (12) and (13), they are α(cosβ+isinβ), α(cosβ−isinβ). 

Thus, the eigenvalues of the compensator subsystem are consistent with that of the interference input variable D(t) and the desired output value $Y^{*}(t)$.

Then, we carry out a transformation of the compensator subsystem in Equations (11–13) as follows:

(14) $$\xi_{1}\left(t+1\right)=\xi_{1}\left(t\right)+\left(\theta K(t)+\lambda L(t)\right)-\left(d_{0}\alpha^{t}\cos\beta t+d_{1}+b\right)$$

(15) $$\xi_{2}\left(t+1\right)=\xi_{1}\left(t\right)+2\alpha \cos\beta \xi_{2}\left(t\right)-\alpha^{2}\xi_{3}\left(t\right)$$

(16) $$\xi_{3}\left(t+1\right)=\xi_{2}\left(t\right)$$

Next, we combine the state equations of the system in Equation (8) with compensator subsystem in Equations (11–13), and a new dynamic system with the following form can be deduced:

(17) $$\left(\begin{array}{l} K(t+1)\\ L(t+1)\\ \xi_{1}\left(t+1\right)\\ \xi_{2}\left(t+1\right)\\ \xi_{3}\left(t+1\right) \end{array}\right)=\left(\begin{array}{ccccc} 1-\delta & 0 & 0 & 0 & 0\\ 0 & 1-\gamma & 0 & 0 & 0\\ \theta & \lambda & 1 & 0 & 0\\ 0 & 0 & 1 & 2\alpha \cos\beta & -\alpha^{2}\\ 0 & 0 & 0 & 1 & 0 \end{array}\right)\left(\begin{array}{l} K(t)\\ L(t)\\ \xi_{1}\left(t\right)\\ \xi_{2}\left(t\right)\\ \xi_{3}\left(t\right) \end{array}\right)+\left(\begin{array}{cc} \mu & 0\\ 0 & \sigma \\ 0 & 0\\ 0 & 0\\ 0 & 0 \end{array}\right)\left(\begin{array}{c} I(t)\\ P(t) \end{array}\right)-\left(\begin{array}{c} 0\\ 0\\ d_{0}\alpha^{t}\cos\beta t+d_{1}+b\\ 0\\ 0 \end{array}\right)$$

Let

(18) $$A=\left(\begin{array}{ccccc} 1-\delta & 0 & 0 & 0 & 0\\ 0 & 1-\gamma & 0 & 0 & 0\\ \theta & \lambda & 1 & 0 & 0\\ 0 & 0 & 1 & 2\alpha \cos\beta & -\alpha^{2}\\ 0 & 0 & 0 & 1 & 0 \end{array}\right),B=\left(\begin{array}{cc} \mu & 0\\ 0 & \sigma \\ 0 & 0\\ 0 & 0\\ 0 & 0 \end{array}\right),Q_{c}=\left(B,AB,\cdots ,A^{4}B\right)$$

For the system in Equation (17), we assign the value of the control vector ${\left(I(t),P(t)\right)}^{T}$ for state feedback as follows:

(19) $$\left(\begin{array}{l} I(t)\\ P(t) \end{array}\right)=F\left(\begin{array}{l} K(t)\\ L(t)\\ \xi_{1}\left(t\right)\\ \xi_{2}\left(t\right)\\ \xi_{3}\left(t\right) \end{array}\right)=\left(\begin{array}{ccccc} k_{1K} & k_{1L} & k_{11} & k_{12} & k_{13}\\ k_{2K} & k_{2L} & k_{21} & k_{22} & k_{23} \end{array}\right)\left(\begin{array}{l} K(t)\\ L(t)\\ \xi_{1}\left(t\right)\\ \xi_{2}\left(t\right)\\ \xi_{3}\left(t\right) \end{array}\right)$$

where

$$F=\left(\begin{array}{ccccc} k_{1K} & k_{1L} & k_{11} & k_{12} & k_{13}\\ k_{2K} & k_{2L} & k_{21} & k_{22} & k_{23} \end{array}\right)$$

is the gain matrix, and $k_{ij},i=1,2,j=K,L,1,\cdots ,3$ are the undetermined coefficients.

If the system in Equation (17) is entirely reachable, i.e., the determinant rank of the controllability matrix $Q_{c}$ of Equation (18) is full, it can be arbitrarily pole allocated by means of substituting the state feedback in Equation (19) into the system in Equation (17). 

Substituting Equation (19) into Equation (17), with the state feedback, the closed-loop system that corresponds to the open-loop system in Equation (17) can be obtained as follows:

(20) $$\left(\begin{array}{l} K(t+1)\\ L(t+1)\\ \xi_{1}\left(t+1\right)\\ \xi_{2}\left(t+1\right)\\ \xi_{3}\left(t+1\right) \end{array}\right)=\left(\begin{array}{ccccc} 1-\delta +\mu k_{1K} & \mu k_{1L} & \mu k_{11} & \mu k_{12} & \mu k_{13}\\ \sigma k_{2K} & 1-\gamma +\sigma k_{2L} & \sigma k_{21} & \sigma k_{22} & \sigma k_{23}\\ \theta & \lambda & 1 & 0 & 0\\ 0 & 0 & 1 & 2\alpha \cos\beta & -\alpha^{2}\\ 0 & 0 & 0 & 1 & 0 \end{array}\right)\left(\begin{array}{l} K(t)\\ L(t)\\ \xi_{1}\left(t\right)\\ \xi_{2}\left(t\right)\\ \xi_{3}\left(t\right) \end{array}\right)-\left(\begin{array}{c} 0\\ 0\\ d_{0}\alpha^{t}\cos\beta t+d_{1}+b\\ 0\\ 0 \end{array}\right)$$

Let

(21) $$A_{c}=\left(\begin{array}{ccccc} 1-\delta +\mu k_{1K} & \mu k_{1L} & \mu k_{11} & \mu k_{12} & \mu k_{13}\\ \sigma k_{2K} & 1-\gamma +\sigma k_{2L} & \sigma k_{21} & \sigma k_{22} & \sigma k_{23}\\ \theta & \lambda & 1 & 0 & 0\\ 0 & 0 & 1 & 2\alpha \cos\beta & -\alpha^{2}\\ 0 & 0 & 0 & 1 & 0 \end{array}\right)$$

If the system in Equation (17) can be arbitrarily pole allocated by means of substituting Equation (19) into Equation (17), then, through an assignment of appropriate value of F, we can allocate the poles of the system in Equation (20). That is, the eigenvalues $z_{i},i=1,\cdots ,5$ of Equation (21) can be allocated as:

(22) $$\left|z_{i}\right|<1,i=1,\cdots ,5$$

If Equation (22) is satisfied, the asymptotic stability control goal can be realized.

As the control variables contain I(t) and P(t), the assignment the proper value of F is the multiple input pole allocation problem. The multiple input pole allocation problem has many solving methods and with a variety of feasible scheme choices, and the pole allocation scheme is not with uniqueness (Zhang et al. [36]).

**Theorem** **1.** *Under the circumstance of the asymptotic stability in system in Equation (20), the compensator subsystem device of Equations (11–13) can realize the control goal, and Y(t) can converge to the desired value:*

(23) $$\underset{t\to \infty }{\lim}Y(t)=Y^{*}(t)$$

The proof of Theorem 1 is provided in Appendix A.

## 4. Numerical Example

With the rapid development, urban population continues to grow resulting in larger domestic water demand including rising tendency. The urban domestic water system demand is also influenced by the seasonal cycle fluctuation. To make the urban domestic water supply adapt to the rising tendency and the seasonal cycle fluctuation of the demand, the water supply industry adjusts production monthly.

In the reference example of the water supply and drainage engineering (Liu et al. [37]), the urban domestic water parameters are set as follows. 

The rate of urban domestic water demand rise a=0.006, thus α=1.006. The one-year period is described as 2π, thus β=π/6. The capital-output factor θ=0.75, the labor-output factor λ=0.25, the rate of capital formation μ=0.85, the rate of capital depreciation δ=0.01, the rate of labor formation σ=0.80, and the rate of labor separation γ=0.005.

According to the above control scheme, we should first calculate the controllability of the system in Equation (17). Substituting the above related parameter values into Equation (18), we have

$$\begin{array}{l} A=\left(\begin{array}{ccccc} 0.9900 & 0 & 0 & 0 & 0\\ 0 & 0.9950 & 0 & 0 & 0\\ 0.7500 & 0.2500 & 1.0000 & 0 & 0\\ 0 & 0 & 1.0000 & 1.7424 & -1.0120\\ 0 & 0 & 0 & 1.0000 & 0 \end{array}\right),B=\left(\begin{array}{cc} 0.8500 & 0\\ 0 & 0.8000\\ 0 & 0\\ 0 & 0\\ 0 & 0 \end{array}\right),\\ Q_{c}=\left(\begin{array}{cccccccccc} 0.8500 & 0 & 0.8415 & 0 & 0.8331 & 0 & 0.8248 & 0 & 0.8165 & 0\\ 0 & 0.8000 & 0 & 0.7960 & 0 & 0.7920 & 0 & 0.7881 & 0 & 0.7841\\ 0 & 0 & 0.6375 & 0.2000 & 1.2686 & 0.3990 & 1.8934 & 0.5970 & 2.5120 & 0.7940\\ 0 & 0 & 0 & 0 & 0.6375 & 0.2000 & 2.3794 & 0.7475 & 5.3943 & 1.6971\\ 0 & 0 & 0 & 0 & 0 & 0 & 0.6375 & 0.2000 & 2.3794 & 0.7475 \end{array}\right) \end{array}$$

Apparently, $\mathrm{rank}\left(Q_{c}\right)=5$, the controllability matrix $Q_{c}$ is non-singular, meeting the sufficient and necessary condition of arbitrary pole allocation of the system in Equation (17) via Equation (19) state feedback.

Then, by means of the undetermined coefficient method, we carry out the pole allocation.

The system matrix $A_{c}$ of Equation (21) contains 10 unknown elements of the gain matrix F. However, there are just five equations necessary for pole allocation of the closed loop system in Equation (20), namely specify the eigenvalue of $A_{c}$ to be $z_{i},i=1,\cdots ,5$. Thus, in the multiple input system pole allocation problem, the number of undetermined elements are more than that of the equations. 

In this problem, there are five undetermined elements of $A_{c}$ that can be freely chosen. Usually, we expect that the elements’ absolute values of the gain matrix F are as low as possible (Wang [38]). Thus, we freely choose the five undetermined elements of F as follows:

(24) $$k_{1L}=k_{13}=k_{2K}=k_{21}=k_{22}=0$$

To realize the control goal with the least delay possible, we specify the eigenvalue of $A_{c}$ as follows:

(25) $$z_{i}=0,\;i=1,\cdots ,5$$

Thus, Equation (25) meets the asymptotic stability prerequisite of Equation (22).

Substituting Equation (24) and related parameters into Equation (21), we solve the following eigenvalue equation

(26) $$\left|zE-A_{c}\right|=0$$

where E is the five-order identity matrix. 

Specifying Equation (25) to be the eigenvalue of Equation (26), we can get the five group solutions as follows:

(27) $$k_{1K}=-6.6085,k_{11}=-21.1575,k_{12}=-28.9631,k_{2L}=1.1122,k_{23}=-18.2631$$

(28) $$k_{1K}=-3.3325,k_{11}=-4.8761,k_{12}=-0.9794,k_{2L}=-2.3685,k_{23}=3.1279$$

(29) $$k_{1K}=-0.8872,k_{11}=-8.5787,k_{12}=23.8856,k_{2L}=-4.9666,k_{23}=-364.5363$$

(30) $$k_{1K}=-3.9504-1.1598i,k_{11}=-4.5611-3.0825i,k_{12}=-0.9347-0.2594i,k_{2L}=-1.7120+1.2323i,k_{23}=2.0011-1.1866i$$

(31) $$k_{1K}=-3.9504+1.1598i,k_{11}=-4.5611+3.0825i,k_{12}=-0.9347+0.2594i,k_{2L}=-1.7120-1.2323i,k_{23}=2.0011+1.1866i$$

As Equations (30) and (31) are a pair of conjugate complex solutions, they are deleted first. Then, we investigate the rest three group real solutions of Equations (27–29). According to the criterion that the elements’ absolute values of the gain matrix F are as low as possible, Equation (28) is reserved as the ultimate solution, and the two other group real solutions are deleted.

Finally, substituting Equations (24) and (28) into Equation (19), the control scheme can be obtained as follows:

(32) $$\left(\begin{array}{l} I(t)\\ P(t) \end{array}\right)=\left(\begin{array}{ccccc} -3.3325 & 0 & -4.8761 & -0.9794 & 0\\ 0 & -2.3685 & 0 & 0 & 3.1279 \end{array}\right)\left(\begin{array}{l} K(t)\\ L(t)\\ \xi_{1}\left(t\right)\\ \xi_{2}\left(t\right)\\ \xi_{3}\left(t\right) \end{array}\right)$$

Equation (32) control scheme can make the urban domestic water system asymptotic stability, and the difference between the supply and demand tends to the desired value. When the urban population grows, seasonal change or other factors lead to all or part of the system parameters α,β,θ,λ,μ,δ,σ,γ, etc. variation; as long as the system in Equation (17) is entirely reachable and Equation (22) is satisfied, the control scheme in Equation (32) will always be effective and able to realize the control goal. Thus, the control scheme has robustness for the parameter changes in the urban domestic water system.

The methodology presented in this study may also be applied to the water management system in other parts of the world, provided all the data used in this study are available.

## 5. Comparison to Existing Models

In the aspect of engineering control decision, some existing models mainly concentrated in the water heater or pump control engineering [2,6,13,20]. In the aspect of economic control decision, in view of the situation that urban water management systems have definite estimating parameters, we constructed some urban water supply and demand investment optimal control models [23,24]. In [23], we considered the urban water demand have the trend of growth and periodicity, and, in [24], we studied town water supply investment control based on dynamic share coefficient method.

However, there are limitations in the two previous models. Firstly, the labor input, an important production factor, is not considered in the models. This will result in the inability to reflect the role of the labor force in achieving the control goal. These previous models used investment as the sole control variables and ignored the role of labor input. However, in general kinds of production functions, labor is usually one of the important factors of production [33]. For this reason, we consider adding labor production factor in our model in this paper. Hence, the control variables of the model consist of both investment and labor. It is a multivariable robust economic control system. This is an improvement of the previous optimal control models, where the latter is just a single variable control system. Compared with the previous optimal control models, the robust model in this study can exhibit the role of the labor force in achieving the control goal.

Secondly, compared with the previous optimal control models, this robust model has greater robustness to the system parameters change. Even in fairly simple problems, the solving and analysis process of the optimal control theory can also be very cumbersome. For this reason, optimal control models often assume that the parameters in the whole planning period remain unchanged. The above optimal control model also keeps the hypothesis of invariance of the system parameters. Affected by demographic factors and climate variables, however, the parameter estimates of urban water supply and demand system may be uncertain and time-varying. Our model in this paper can handle system parameter uncertainties and time-varying by using robust control method, so it can overcome the disadvantages of the above optimal control model quite well.

We continue the numerical example in Section 4 to illustrate the validation of our model. Assuming that under the influence of demographic factors and climate variables, some parameters change in the different time during the control period as follows:

α=1.008,θ=0.7,λ=0.3,μ=0.8,σ=0.85,γ=0.006

α=1.004,θ=0.8,λ=0.2,μ=0.9,σ=0.6,γ=0.003

α=1.002,θ=0.75,λ=0.25,μ=0.65,σ=0.7,γ=0.005

α=1.005,θ=0.8,λ=0.2,μ=0.65,σ=0.7,γ=0.005

Next, we examine whether the original control scheme in Equation (32) is still effective. 

Through calculations, the system in Equation (17) is entirely reachable in all these cases; and all of the eigenvalues $z_{i},i=1,\cdots ,5$ of Equation (21) corresponding to these cases are as follows:

$$\left(\begin{array}{ccccc} 0.8607 & 0.2389+0.8106i & 0.2389-0.8106i & -0.7723 & -0.5155 \end{array}\right)$$

$$\left(\begin{array}{ccccc} -0.5274+0.8557i & -0.5274-0.8557i & 0.5975+0.5611i & 0.5975-0.5611i & 0.1654 \end{array}\right)$$

$$\left(\begin{array}{ccccc} 0.2051+0.9304i & 0.2051-0.9304i & -0.5422 & 0.5142+0.2272i & 0.5142-0.2272i \end{array}\right)$$

$$\left(\begin{array}{ccccc} 0.0896+0.9976i & 0.0896-0.9976i & 0.6563+0.4866i & 0.6563-0.4866i & -0.5902 \end{array}\right)$$

Thus, Equation (22) is satisfied. Therefore, the control scheme in Equation (32) remains effective in all these cases.

For the last case above, if the labor formation σ continues increasing, parameters change as follows:

α=1.005,θ=0.8,λ=0.2,μ=0.65,σ=0.8,γ=0.005

Through calculations, the system in Equation (17) is still entirely reachable in the case, but all of the eigenvalues $z_{i},i=1,\cdots ,5$ of Equation (21) in the case are as follows:

$$\left(\begin{array}{ccccc} -0.8749 & 0.0891+1.0285i & 0.0891-1.0285i & 0.6807+0.4799i & 0.6807-0.4799i \end{array}\right)$$

As the second and the third poles are located outside the unit circle, Equation (22) is not satisfied. Therefore, the control scheme in Equation (32) cannot guarantee the system is asymptotically stable in this case. Hence, the control scheme needs to be adjusted. 

The adjusted scheme is quite simple. Through calculations, we only need to slightly increase the coefficient $k_{2L}$ of the gain matrix F of Equation (32). Let $k_{2L}=-1.9685$, and all the other coefficients of the gain matrix F of Equation (32) remain the same. 

The system in Equation (17) is also entirely reachable in the adjusted scheme and all of the eigenvalues $z_{i},i=1,\cdots ,5$ of Equation (21) in the adjusted scheme are as follows:

$$\left(\begin{array}{ccccc} 0.1411+0.9900i & 0.1411-0.9900i & 0.5734+0.3718i & 0.5734-0.3718i & -0.4443 \end{array}\right)$$

Thus, Equation (22) is satisfied. Consequently, the adjusted control scheme can make the system asymptotically stable again. 

For the compared optimal control models [23,24], however, due to the limitations of system parameter invariance hypothesis, they cannot handle all the above situations of parameter changes. Thus, the robust model in this paper has stronger adaptability than the compared optimal control models.

## 6. Conclusions

Along with China’s rapid urbanization, urban domestic water generally presents the circumstances of both rising tendency and seasonal cycle fluctuation. The multiplicative model is appropriate for describing the urban domestic water demand function. To meet the urban domestic water demand, we use investment and labor as control variables in the water supply. Through the compensator design and pole allocation, the implementation of multivariable robust economic control decision method can realize the urban domestic water supply control goal, and it has robustness for the variation of parameters. When the parameters of the system are available, this method may also be applied to the water management system in other parts of the world. Besides the urban domestic water supply system, the robust control method in this paper is also applicable to deal with tracking control problems or stabilization control problems of other general dynamic uncertain systems.

Finally, we should point out some limitations of this study. One is the lack of real water supply system to illustrate the model developed in this paper and to validate the effectiveness of the model. Another limitation is that the technical level plays a positive role in improving productivity, and some production functions contain the technical level as a variable; however, the model developed in this study does not include the technology level. In future model improvement, we may consider including more production factors in the model.

## Appendix A

**Proof** **of** **Theorem** **1.** When we substitute Equation (19) into Equation (17), making the system in Equation (17) to be arbitrary pole allocation, the system can be asymptotically stable if Equation (22) is satisfied. When t is sufficiently large under the circumstance, due to the system asymptotic stability, the free movement no longer exists in the system, only the force movement impacts on the system state. The eigenvalues of the *Z*-transform of the interference input variable D(t) are α(cosβ+isinβ),α(cosβ−isinβ),1, the eigenvalue of the *Z*-transform of the desired output value $Y^{*}(t)$ is 1, and the signal form of D(t) contains that of $Y^{*}(t)$. Therefore, the signal form of all the state variables of the system in Equation (20) will be consistent with that of D(t). The following limits exist:

(A1) $$\underset{t\to \infty }{\lim}K(t)=\xi_{K1}\alpha^{t}\cos\beta t+\xi_{K2}$$

(A2) $$\underset{t\to \infty }{\lim}L(t)=\xi_{L1}\alpha^{t}\cos\beta t+\xi_{L2}$$

(A3) $$\underset{t\to \infty }{\lim}\xi_{1}(t)=\xi_{11}\alpha^{t}\cos\beta t+\xi_{12}$$

(A4) $$\underset{t\to \infty }{\lim}\xi_{2}(t)=\xi_{21}\alpha^{t}\cos\beta t+\xi_{22}$$

(A5) $$\underset{t\to \infty }{\lim}\xi_{3}(t)=\xi_{31}\alpha^{t}\cos\beta t+\xi_{32}$$

where $\xi_{ij},i=K,L,1,\cdots ,3,j=1,2$ are constants. When t is sufficiently large, derived from Equation (A5), we have

(A6) $$\xi_{3}(t+1)=\xi_{31}\alpha^{t+1}\left(\cos\beta t\cos\beta -\sin\beta t\sin\beta \right)+\xi_{32}$$

(A7) $$\xi_{3}(t+2)=\xi_{31}\alpha^{t+2}\left(\cos\beta t\cos2\beta -\sin\beta t\sin2\beta \right)+\xi_{32}$$

Substituting Equations (A3) and (A5)–(A7) into Equation (13), we get

(A8) $$\xi_{31}\alpha^{t+2}\left(\cos\beta t\cos2\beta -\sin\beta t\sin2\beta \right)+\xi_{32}-2\alpha \cos\beta \left(\xi_{31}\alpha^{t+1}\left(\cos\beta t\cos\beta -\sin\beta t\sin\beta \right)+\xi_{32}\right)+\alpha^{2}\left(\xi_{31}\alpha^{t}\cos\beta t+\xi_{32}\right)=\xi_{11}\alpha^{t}\cos\beta t+\xi_{12}$$

According to the trigonometric function Equation,

(A9) $$\sin2\beta =2\sin\beta \cos\beta ,\cos2\beta =2\cos^{2}\beta -1$$

and substituting Equation (A9) into Equation (A8), we get

(A10) $$\xi_{11}\alpha^{t}\cos\beta t+\xi_{12}+\left(2\alpha \cos\beta -\alpha^{2}-1\right)\xi_{32}=0$$

Since Equation (A10) holds for all t, there must be

(A11) $$\xi_{11}=0$$

Substituting Equation (A11) into Equation (A3), we get

(A12) $$\xi_{1}(t)=\xi_{12}$$

which shows that $\xi_{1}(t)$ is a constant. Substituting Equation (A12) into Equation (11), we obtain

(A13) $$Y\left(t\right)-Y^{*}\left(t\right)=0$$

Therefore, Equation (23) is proved. ☐
